# Recent Insights into Antibiotic Resistance in *Helicobacter pylori* Eradication

**DOI:** 10.1155/2012/723183

**Published:** 2012-07-05

**Authors:** Wenming Wu, Yunsheng Yang, Gang Sun

**Affiliations:** Department of Gastroenterology and Hepatology, Chinese PLA General Hospital, No. 28 Fuxing Road, Beijing 100853, China

## Abstract

Antibiotics have been useful in the treatment of *H. pylori*-related benign and malignant gastroduodenal diseases. However, emergence of antibiotic resistance often decreases the eradication rates of *H. pylori* infections. Many factors have been implicated as causes of treatment failure, but the main antibiotic resistance mechanisms described to date are due to point mutations on the bacterial chromosome, a consequence of a significantly phenotypic variation in *H. pylori*. The prevalence of antibiotic (e.g., clarithromycin, metronidazole, tetracycline, amoxicillin, and furazolidone) resistance varies among different countries; it appears to be partly determined by geographical factors. Since the worldwide increase in the rate of antibiotic resistance represents a problem of relevance, some studies have been performed in order to identify highly active and well-tolerated anti-*H. pylori* therapies including sequential, concomitant quadruple, hybrid, and quadruple therapy. These represent a promising alternatives in the effort to overcome the problem of resistance. The aim of this paper is to review the current status of antibiotic resistance in *H. pylori* eradication, highlighting the evolutionary processes in detail at alternative approaches to treatment in the past decade. The underlying resistance mechanisms will be also followed.

## 1. Introduction

*Helicobacter  pylori* is a spiral-shaped, microaerophilic Gram-negative flagellate bacterium that may contribute to diseases such as duodenal/gastric ulcer disease, gastritis, gastric adenocarcinoma, and mucosa-associated tissue lymphoma (MALT) and primary B-cell gastric lymphoma. Given this relationship with human diseases, eradication of *H. pylori* in individuals may be the best course of action. In fact patients who receive *H. pylori* eradication therapy (proton pump inhibitor (PPI), amoxicillin (AMO), and clarithromycin (CLA)) often encounter eradication failure over their treatment period. Moreover, the effectiveness of “legacy triple therapy” which was recommended by Maastricht III Consensus Report provides disappointingly low treatment success (i.e., below 80%) in the world. And what could account for the resulting low treatment success or eradication failure? The reasons for this fall in effectiveness are uncertain but may be mainly related to the development of antibiotic resistant strains of *H. pylori*. In this paper, we will review the latest findings on *H. pylori* and antibiotic resistance and then summarize the factors for *H. pylori* eradication failure according to the current treatment regimens.

## 2. Nature of *H. Pylori* and Intragastric Environment

**Figure 1:**
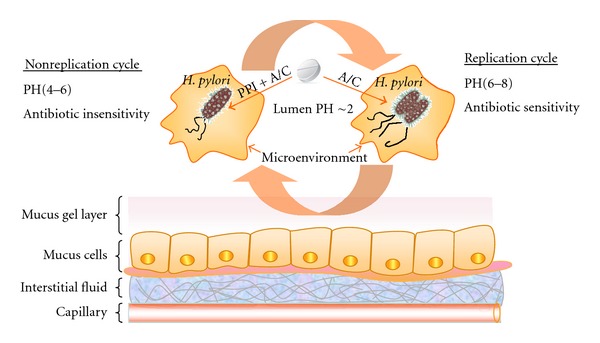
*H. pylori* oscillates between a replicating state (antibiotic sensitivity) and nonreplicating state (antibiotic insensitivity) according to the pH in the microenvironments, and PPI synergizes with the antibiotics by effectively increasing gastric pH and disrupts the acidic environment preferred by HP. PPI: proton pump inhibitor, A: amoxicillin, C: clarithromycin.

The stomach environment where the *H. pylori* resides was thought to be a virtual desert for microbes because of its high acidity. We now know *H. pylori* dominates the microbiome in the stomach, although the effect of this dominance is unclear [1]. A major opportunity to increase our understanding of this microbiome is massive parallel pyrosequencing of bacteria 16S amplicons. This will allow us to deeply characterize the microbiota of a wide range of subjects [2]. One such study used this small subunit 16S rDNA clone library to analyze 1833 sequences generated by broad-range bacterial PCR from 23 gastric endoscopic biopsy samples. This data suggests that *H. pylori* was the only member of the genus *Helicobacter* found in these human stomach samples and was the most abundant phylotype within the libraries which tested positive for this organism by using conventional clinical approaches [3]. The huge population of *H. pylori* is also the statistical basis of existing population of resistant organisms [4]. In addition, the bacteria oscillates between a replicating state (organism remains susceptible to the antibiotic) and nonreplicating state (the organism become phenotypically resistant) according to the pH in the microenvironments. Thus, they may enter to a nonreplicative but viable state when the pH around their microenvironments is between 4.0 and 6.0. These organisms will be difficult to eradicate, in other words, if they present the phenotypically resistant state [5] (Figure 1).

*H. pylori* infection also presents a unique therapeutic challenge. Determining the optimal drug therapy of such infection depends to a large extent on antimicrobial concentrations in the stomach, while it is difficult because the organism lives in an environment that is not easily accessible to some medications [6]. Upon entry into the stomach, the first hurdle for bioavailability of antibiotics is the acidity of the gastric lumen, which in humans has a median 24 h intragastric pH of 1.4 [7]. A good example of this is one of the most acid-labile antibiotics against *H*. *pylori*, such as clarithromycin (CLA), which is degraded in the lumen mainly through the action of acid and pepsin. Its half life is less than 1 h in this circumstance. It became clear early on that antibiotic treatment alone was relatively ineffective. Thus, increasing intragastric pH by the coadministration of potent gastric acidity inhibitors has been shown to significantly avoid eradication failure [8]. The second hurdle is the particular structure of gastric mucus. To successfully kill the bacteria present in the stomach it is necessary that the drug is delivered to the entire surface of the stomach and penetrates across the mucus layer from gastric lumen to epithelial surface (or vice versa); furthermore, the antibiotic must reach higher concentrations for a sufficient time to efficiently kill the bacteria wherever they are present [9]. Otherwise, the bacteria in such sites can recolonize the gastric epithelium, resulting in eradication failure [10]. Significant work should be undertaken in an attempt to overcome the gastric barrier, including developing several strategies to target either the transcellular or the paracellular pathway for drug delivery.

## 3. Epidemiology of Bacterial Resistance

**Figure 2:**
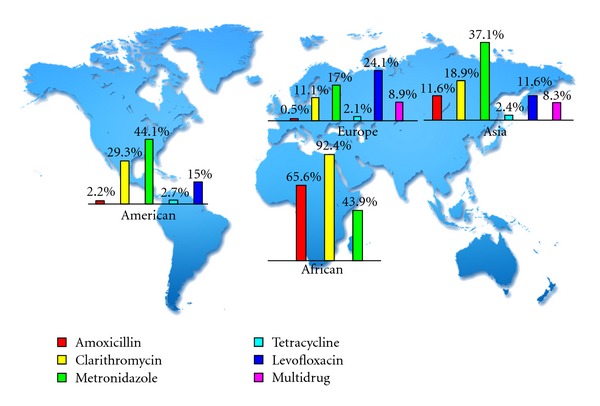
Antibiotic resistance rates in different continental areas.

It is now believed that some populations with high incidences of *H*. *pylori* infection, such as those in East Asian countries, have high incidences of gastric cancer, while other highly infected populations do not. This apparent anomaly has been termed the “African enigma” or “Asian enigmas”. It might be explained by diverse the *H*. *pylori* genotypes, especially cagA and vacA, circulating in different geographic areas [11, 12]. Like the *H*. *pylori* infection associated with geographic areas, the prevalence of resistance rates appears to be partly determined by geographical factors; the prevalence of CLA and metronidazole (MET) resistance in China both increased from 12.8 to 23.8% and, 12.8 to 56.6%, respectively, while AMO resistance decreased from 2.1% to 0.3%, between 2000 to 2009 [13]. In Japan, adverse resistance rates to CLA increased from 7% to 15.2%, and the rate has remained fairly constant to the present day [14]. A high resistance of MET has been reported from Saudi Arabia. The rate of resistance to MET in 2008 was 69.5%, while CLA and AMO resistance rates were 21% and 0%, respectively [15]. In Europe, there are huge differences between southern and northern Europe. Higher resistance rates of clarithromycin in adults are observed in southern European countries such as Spain where the rate of CLA resistance was 35.6% in patient isolates of *H*. *pylori* [16]. Generally speaking, it was as high up as 20% compared to northern European countries [17, 18]. CLA resistance is seemingly common in the USA, ranging 10–15%, while MET resistance rates are 20–40% and resistance to amoxicillin appears to be infrequent [19, 20]. Mendonça et al. analyzed 90 Brazilian dyspeptic patients and revealed that resistance of *H*. *pylori* to clarithromycin, metronidazole, tetracycline (TET), amoxicillin (AMO), and furazolidone (FUR) was 7%, 42%, 7%, 29%, and 4%, respectively [21]. A meta-analyses reported the overall *H*. *pylori* antibiotic resistance rates worldwide (31 studies from 1993 to 2009) which showed an overall *H*. *pylori* antibiotic resistance rate for AMO, CLA, MET, TET, levofloxacin (LEV), and multidrug-based therapies in different continental areas [22]. Detailed resistance rates towards antibiotics in different continental areas are shown in Figure 2.

Some of the reasons for these findings may include the following (1) CLA was widely administered as monotherapy for respiratory infections and as a consequence high resistance rates are reported in these countries [23]. (2) The prevalence of antibiotic resistance in various regions is correlated with the general use of antibiotics in the region, while countries with a prudent consumption of macrolides continues to be low [24]. (3) *H. pylori* strains have been divided into five major groups (east Asian type, south/central Asian type, Iberian/African type, and European type) according to geographical associations [25]. Thus, geographic differences associated with the presence of phylogeographic features of *H. pylori* may be a factor to explain the existing different antibiotic resistances [26, 27].

## 4. Current Anti-*H. Pylori* Regimens

**Figure 3:**
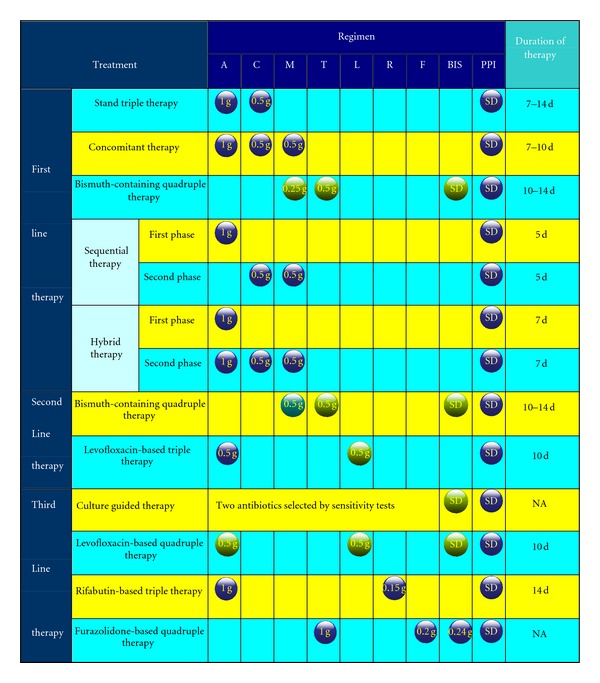
Current recommended regimens for *H*. *pylori* eradication. The figure in the ball stands for dose. Blue ball: b.i.d, purple ball: t.i.d, green ball: q.i.d. A: amoxicillin, C: clarithromycin, M: metronidazole, T: tetracycline, L: levofloxacin, R: rifabutin, F: furazolidone, SD: standard dose, BIS: bismuth, PPI: proton pump inhibitor, modified from [31–41].

*H. pylori* eradication therapy, including antibiotics, PPI, and/or bismuth given for one or two weeks, has emerged as the treatment of choice (Figure 3). Standard triple therapy which represents the accepted standard therapy for *H. pylori* is known to be susceptible to clarithromycin, and local antimicrobial resistance rates are below to 20% [28], while newer treatment regimens (sequential, quadruple, concomitant, and hybrid therapies) and various combinations of new and old antibiotics aimed at eradicating the organism more effectively are increasing in popularity [29, 30].

**First Line Therapy**

As first-line therapy in areas with a high prevalence of clarithromycin-resistant *H. pylori* strains, a novel 10 d sequential therapy should be considered. The sequential regimen containing a dual therapy (PPI and amoxicillin for 5 days) was followed by triple therapy with a PPI, clarithromycin, and tinidazole (or metronidazole) for 5 days. The eradication rate achieved with the sequential regimen has been reported significantly greater than that obtained with the standard treatment [32, 42]. However, it has shown that sequential therapy is ineffective in clearing *H. pylori* in patients with dual resistance to clarithromycin and metronidazole [23, 33]. Another new regimen term as concomitant therapy is a 4-drug non-bismuth-containing regimen (PPI, clarithromycin, amoxicillin, and metronidazole), which appears more suitable for patients in high endemic areas of dual resistance. Clinically, it is also more simple than sequential therapy as the drugs are all given together instead of changing drugs in halfway and might improve compliance. In addition, an intention-to-treat analysis demonstrated sequential or concomitant therapy with a PPI, amoxicillin, clarithromycin, and an imidazole agent has similar rates for eradication of *H. pylori* infection [42]. With regard to dual resistance, several attempts, such as the extension of sequential therapy duration and continuing the amoxicillin for the full 14 days of therapy, have been undertaken to improve the efficacy of the standard PPI triple therapy. Recently, a sequential-concomitant hybrid therapy (dual-concomitant) was designed by Hsu et al. [43]. The date showed that it provides a promising success rate of 99% by per-protocol analysis and 97% by intention-to-treat analysis. However, it must be noted that it may not work in all geographic areas, and the results will need to be confirmed in areas where different patterns of resistant are present.

**Second Line Therapy**

Bismuth-containing quadruple therapy as second-line and/or salvage therapy was recommended by Maastricht IV/Florence Consensus Report [34]. Several multicenter studies of quadruple therapy using a single-triple (bismuth biskalcitrate, metronidazole, and tetracycline) capsule preparation with PPI have shown good efficacy for eradication of *H*. *pylori* [35, 36, 44]. Convenience packs that contain most of drugs in a plasticized sheet also reduce the number of pills to improve adherence. As for adverse effects, toxic effects related to bismuth are still one of the unjustified safety concerns against the quadruple therapy [37], thus, we needed to establish the reasonable bismuth dosing regimen that provides maximum eradication.

In patients who failed with clarithromycin-based triple in first line, levofloxacin-based triple therapy (levofloxacin, amoxicillin, and a PPI) has been proven in a meta-analysis which showed that this regimen was superior to quadruple therapy and fewer side effects as salvage therapy [45]. Additionally, the study revealed that antibiotics (i.e., levofloxacin) within this triple regimens cannot randomly be changed and then switched to first line. For antibiotic resistance, rising rates of levofloxacin resistance especially in developing countries remain to be taken into account, and it appears more likely that quinolone resistance is usually relating to patients who have routinely received a fluoroquinolone for other indications [38].

**Third-Line Therapy**

To date, the standard third-line therapy for refractory *H. pylori* infection has not been established. Maastricht IV reports recommend that anti-*H*. *pylori* treatment should be guided by antimicrobial susceptibility testing after failure of second-line therapy, whenever possible [34]. Unfortunately, antimicrobial sensitivity data for patients who failed eradication therapy is still not widely available in clinical practice. For practitioners, several simple empirical management strateges are necessary.

A recent prospective study assesses the efficacy and safety of levofloxacin, amoxicillin, bismuth, and rabeprazole quadruple therapy as third-line treatment for patients who failed to eradicate *H. pylori* infection. In this investigation, the 10-day levofloxacin and amoxicillin-based quadruple rescue therapy provides superior eradication with an additional clinically important benefit of improved tolerability due to fewer side effects [30]. Other alternative candidates for third-line therapy are rifabutin; quinolones therapy is also promising [39, 40, 46], though the optimal dose and combination need further study.

## 5. Antibiotic Resistance Mechanisms in the Current Regimens

As a general rule for the treatment, it is defined on meeting or exceeding predefined per-protocol threshold cure rates (e.g., >90%), that is, eradication failure less than 10% [47]. *H. pylori*'s antimicrobial resistance rates vary as mentioned above. *H. pylori* eradication failures may be due to acquiring chromosomal mutations or by acquisition of foreign genes carried on mobile genetic elements (horizontal gene transfer) that cause changes in each drug's site of action [23, 48], and it cannot be reversed by increasing the dose or duration [41]. Each of these mechanisms will be elucidated in more detail below according to the current anti-*H. pylori* regimens.

**Clarithromycin**

In a recent study involving sequencing analysis of *H. pylori* gene 23S *rRNA* isolated from Uruguayan patients, all CLA-resistant strains point mutation were presented in position 2143 (A-to-G transition), consistent with strains studied in some developing countries worldwide. No AMO-resistant strains were identified in this study, this is most frequently reported with AMO where failure is rarely caused by acquired resistance [49, 50]. Other mutations at position 2142 (A-to-G transition) and position 2182 (C-to-T transition) have been confirmed by analysis of DNA sequencing to be the same as that described at position 2143 and are associated with CLA resistance [51]. Except for 23S *rRNA* mutations, expression of a resistance-nodulation-cell division (RND) type efflux pump, an active drug efflux mechanism responsible for rapidly transferring the drug out of the bacterial cell, preventing the binding of the antibiotic to the ribosome, plays an important role in acquiring CLA resistance [52, 53]. Nevertheless, it was shown that efflux systems are not involved in the intrinsic resistance of *H. pylor*i to antibiotics or in acquired resistance to AMO [54].

**Amoxicillin**

Rare tolerance to AMO has also been described and was associated to alterations in penicillin binding proteins (PBP1A) [55]. Three substitutions (Ser 414 Arg, Thr 556Ser, and Asn 562) are the most common amino acid changes in PBP1 connected to AMO resistance. Consequently, this reduces the susceptibility of these strains to the bactericidal effect of AMO [56].

**Metronidazole**

Metronidazole (MET) resistance in *H. pylori* is complex and is primarily associated with mutational inactivation of the redox-related gene (*frxA*, *rdxA*) [57]. *FrxA* may act indirectly by affecting cellular reductive potential in low level MET resistant isolates. *RdxA* gene inactivation confers resistance by saturation transpose on mutagenesis of the *H. pylori* genome [58, 59]. Thus, factors that lead to the loss of or inactivation of the two genes may lead to contribute to MET resistance per se. Meanwhile, there are reports that the MET resistance phenotype may arise in *H. pylori* without mutations in *rdxA* or *frxA*, suggesting the presence of additional MET resistance mechanisms [60]. Choi et al. proposed that several mutational changes in* H. pylori* Fur proteins can affect MET susceptibility via altering the balance among Fur's several competing activities and thereby eliminating bactericidal MET activation products [61].

**Fluoroquinolone**

The mechanism of fluoroquinolone (FLU) resistance in *H. pylori* has been found to be linked to mutations in the quinolone resistance-determining regions (QRDR) of the gyrase A (*gyrA*) gene [62]. This region, responsible for DNA cleavage and rearrangement, is also the position of action of quinolones [39]. A recent study performed in Korea has shown this resistance was considered to depend mainly on *gyrA* gene mutation at Asn87 or Asp91 [63], and mutation in the *gyrB* gene has also been identified in LEV resistant strains. This rarely occurs and often occurs together with *gyrA* mutations. This indicates that *gyrB* has little impact on primary levofloxacin resistance. In addition, *gyrA* gene has double *gyrA* mutations hot spots at N87K and D91G or D91Y which were linked to high-level fluoroquinolone resistance by laboratory mutants [64].

**Rifabutin**

Rifabutin (RIF) is a spiropiperidyl rifamycin-S derivative, which inhibits the B-subunit of the DNA-directed *RNA* polymerase (*rpoB*) of *H. pylori*. RIF has potential activity against *H. pylori* because the *in vitro* sensitivity is high, and it does not share resistance to either CLA or AMO [65, 66]. It is structurally related to rifampin (rifampicin) and shares many of its properties. The mechanism of *H. pylori* resistance to this group of antibiotics is not known, only some studies clearly show that it is substantial cross-resistance *in vitro* between rifabutin and rifampin, mainly caused by point mutations occurring in the *rpo*B gene at codons 524, 525, and 585 as in other bacteria [66–68].

**Tetracycline**

Tetracycline (TET) is an antibiotic that is commonly used to eradicate *H. pylori* infection in several second-line regimens. The bactericidal activity of TET is a result of the drug's ability to prevent the synthesis of nascent peptide chains via binding to the 30S ribosomal subunit as well as blocking the binding of aminoacyl-*tRNA* [69]. The best-studied resistant mechanism has been mostly associated with de novo mutations in the 16S *rRNA* gene, which is based on a single, double or triple base-pair substitution in adjacent 16S *rRNA* gene [70]. In the case of mutation that cause resistance, single or double base-pair substitutions (A928C, AG926-927→GT and A926G/A928C) as well as triple substitution (AGA926–928→TTC) confer *H. pylori* with low and high-level TET resistance [71]. The phenotype observed in the case of this mutant is similar to those observed by Gerrits et al. [72]. Probably, decreased antibiotic binding of the drug for the ribosome reduces its antibiotic property. Resistance to TET is also related to a proton motive force (PMF)-dependent efflux of TET across the cell membrane. Consistent with efflux studies, carbonyl cyanide m-chlorophenylhydrazone (CCCP), an inhibitor that disrupts the proton gradient across the membrane, leads to antibiotic accumulation by presence or absence of it. Therefore, it plays an important role in the resistance of clinical isolates of *H. pylori* to TET [73].

## 6. Conclusion

*H. pylori* is considered pathogenic, even carcinogenic. With this simple view, eradication is considered as an obvious choice. In reality, however, the rate of eradication failure has dramatically risen in many countries due to resistance to antibiotics. On genetic support, mutation is considered as the key phenotypic variation as well as response to selection stress. Other suspected mechanisms of acquired drug resistance include: decreased permeation of the antibiotic into the bacterial cell and multidrug efflux pumps confer resistance to *β*-lactams [54]. An opportunity to solve this is whole-genome sequencing of multiple isolates of individual patients with dense spatial and temporal sampling. A practical application is the detection of genomic changes related to drug resistance by comparing the genomes of wild-type strains and those that survived antibiotic treatments [74, 75]. Furthermore, in the context of clinic treatment, selection pressure exerted by the long-term use of antibiotics, drug adverse effect, patient tolerability, adherence, even the patient's disease status should considered by doctors [4]. It is important to remember that antibiotic resistance can often be partially overcome by susceptibility and DNA testing and differentiation of recrudescence and reinfection. Highly active and well-tolerated regimen should be sought and appropriately tested in randomized controlled trial (RCT) instead of simply following consensus guidelines.
